# Notch Signalling Controls Differential Proliferation During Rotation and Asymmetric Morphogenesis of Mouse Incisors

**DOI:** 10.3390/cells15211947

**Published:** 2026-10-26

**Authors:** Argyro Lamprou, Cristina Porcheri, Thimios A. Mitsiadis

**Affiliations:** 1 Institute of Oral Biology, Centre of Dental Medicine, Faculty of Medicine, University of Zurich, 8032 Zurich, Switzerland; cristina.porcheri@zzm.uzh.ch; 2 Foundation for Research and Technology-Hellas (FORTH), University of Crete, 700 13 Heraklion, Greece; 3 Center of Excellence in Precision Medicine and Digital Health, Department of Physiology, Faculty of Dentistry, Chulalongkorn University, Bangkok 10330, Thailand

**Keywords:** incisor, tooth, Notch signalling, cell proliferation, cell cycle, transgenic mice

## Abstract

Rodent incisors exhibit pronounced labial–lingual asymmetry, with enamel deposited exclusively on the labial surface and cementum on the lingual surface. This asymmetry arises early in development as the initially symmetrical dental epithelium rotates and reorients along the jaw axis. Here, we investigated the cellular and molecular mechanisms underlying this process. Quantitative cell-cycle analyses and transcriptomic profiling before and during epithelial rotation revealed accelerated proliferation, shorter cell-cycle duration, and enrichment of DNA replication and mitotic genes within the labial epithelium compared with the lingual epithelium. The evolutionarily conserved Notch signalling pathway, a regulator of cell proliferation and fate, was asymmetrically active during this period, with Notch2 expression restricted to the labial domain. Lineage tracing demonstrated that Notch2-expressing cells and their progeny preferentially contribute to the enamel-producing compartment. Epithelial-specific deletion of Notch2 produced no obvious early morphological phenotype and induced upregulation of other Notch pathway components. By contrast, pharmacological Notch inhibition reduced epithelial asymmetry and altered S-phase progression, and decreased Ccnd1 and Cdk6 expression. Together, these findings implicate Notch signalling in regional cell-cycle dynamics during early mouse incisor asymmetry, while suggesting that other Notch components may compensate for Notch2 loss.

## 1. Introduction

Morphogenesis of ectodermal appendages, including hairs, mammary glands, salivary glands, and teeth, proceeds through highly conserved programmes of reciprocal epithelial–mesenchymal interactions governed by shared molecular regulators [1,2,3]. Among these organs, rodent incisors are distinguished by their capacity for continuous growth and by a pronounced labial–lingual asymmetry, with enamel deposited exclusively on the labial surface and cementum on the lingual surface [4,5]. This asymmetry is established early in embryogenesis, when the initially symmetrical epithelial bud undergoes rotation along the jaw axis to generate distinct labial and lingual compartments [6,7]. Although the morphological progression of incisor development has been extensively characterised, the cellular and molecular mechanisms that drive asymmetric epithelial growth remain poorly understood.

Notch signalling is an evolutionarily conserved pathway that regulates proliferation, differentiation, and fate specification across diverse tissues [8]. In mammals, the pathway comprises four receptors (Notch1-4) and five ligands (Jagged1, Jagged2, Delta-like ligand 1, 3, and 4) [9,10]. Ligand-receptor binding triggers sequential proteolytic cleavages that release the Notch intracellular domain (NICD), which translocates to the nucleus and activates transcriptional targets including *Hes1*, *Hes5*, *Hey1*, and *Hey2* [9]. During tooth development, Notch signalling contributes to multiple aspects of morphogenesis and dental epithelial identity [11,12,13,14,15,16,17,18]. Notably, several Notch pathway components exhibit asymmetric expression within the incisor epithelium before bud rotation, raising the possibility that Notch signalling contributes to the establishment of epithelial asymmetry [6,7]. However, whether Notch activity functionally regulates asymmetric growth during incisor morphogenesis remains unknown.

Here, we combined quantitative morphometry, cell-cycle analysis, transcriptomic profiling, and genetic and pharmacological perturbations to investigate early morphogenesis of the mouse mandibular incisor. The results support a model in which regional differences in epithelial cell-cycle dynamics contribute to labial expansion and incisor asymmetry. Notch2 deletion alone did not produce an evident early phenotype, whereas pharmacological Notch inhibition reduced the labial-lingual area difference and altered cumulative EdU incorporation. These findings implicate Notch signalling as a key regulator of incisor epithelial asymmetry, but they do not establish that the same mechanism operates in other teeth or appendages.

## 2. Materials and Methods

### 2.1. Mouse Strains and Ethical Statement

C57BL/6J wild-type mice and *Keratin14-Cre/+;Notch2fl/fl*, *Keratin14-Cre/+;R26mT/mG*, and *Notch2-CreERT2/+;R26mT/mG* mutant mice were used between embryonic days (E) 12.5 and 14.5 [19,20,21]. Mice were maintained under standard specific pathogen-free conditions at the Laboratory Animal Services Center, Zurich. All animal experiments were approved by the cantonal veterinary office in Zurich (licenses #203/2020, active since 07.01.2022 and #195/2023, active since 11.04.2024) and performed in accordance with the Swiss animal welfare act. Embryonic stages were determined by vaginal plug detection (E0.5) and confirmed by morphological criteria. Pregnant females at E13–E14 received intraperitoneal injections of 5-ethynyl-2′-deoxyuridine (EdU; ABP Biosciences, Rockville, MD, USA, #ABP-A012) and/or 5-bromo-2′-deoxyuridine (BrdU; Thermo-Fischer Scientific, Waltham, MA, USA, #B23151) at 50 mg/kg body weight. Embryos were collected 1 h after BrdU injection for proliferation assays. In the current study, only lower incisors were included in the analysis.

### 2.2. Orientation and Regional Definition

**Figure 1:**
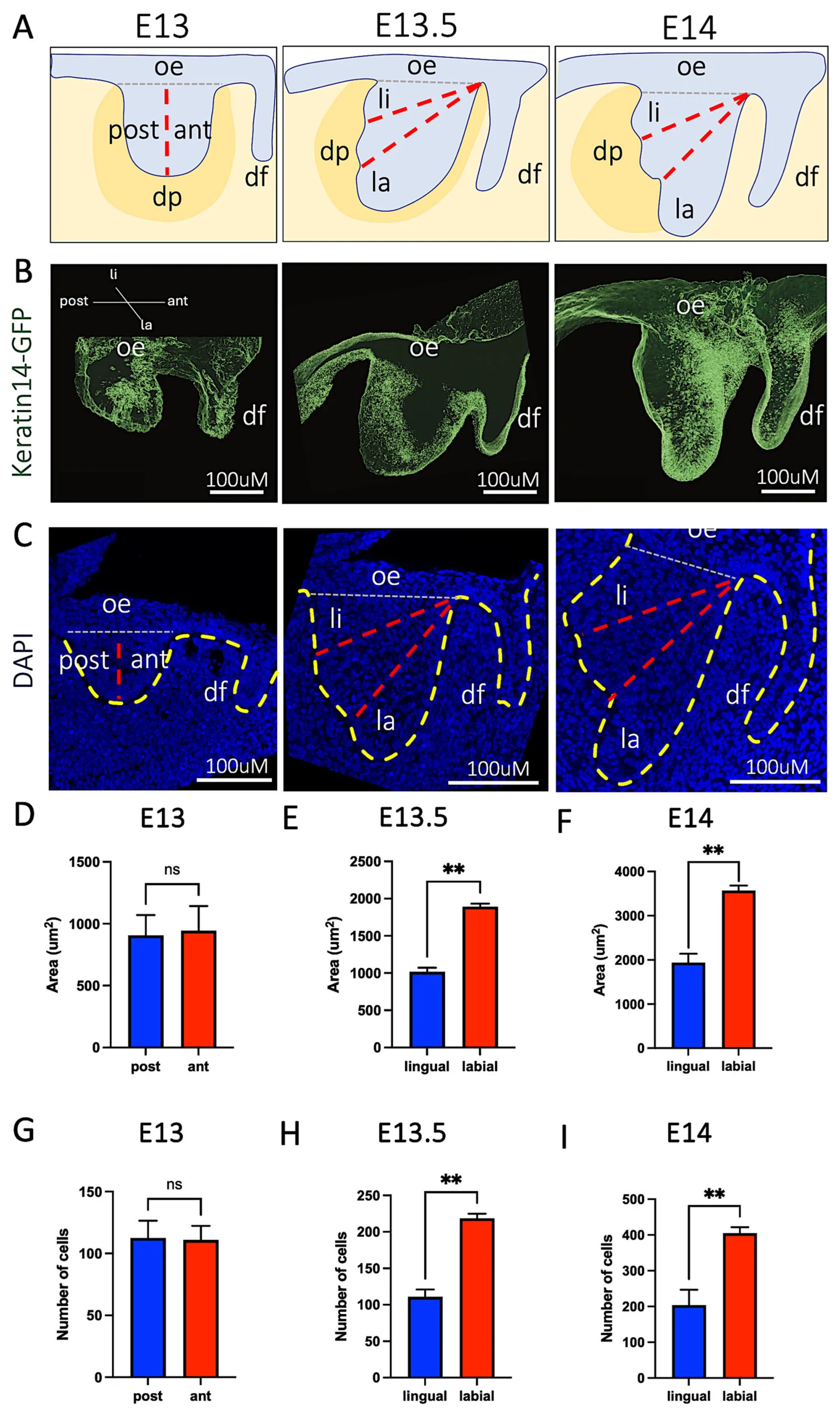
Morphological changes in the incisor epithelium during the establishment of asymmetry. (**A**) Schematic of embryonic day 13–14 (E13–E14) incisor germs. The incisor epithelium is shown in blue and the surrounding mesenchyme in yellow. Red dashed lines define the boundary between anterior-posterior (E13) and labial-lingual epithelia (E13.5 and E14). Grey dashed lines indicate the borders of the oral epithelium. (**B**) Surface-rendered segmentation of whole-mount immunofluorescence images from *Keratin14Cre/+;R26mT/mG* incisor germs at E13–E14 showing epithelial morphology during asymmetry establishment. (**C**) Immunofluorescent staining of E13–E14 incisor sections. Nuclei are labelled with DAPI. Yellow dashed lines outline the epithelium and red dashed lines delineate the anterior–posterior and labial–lingual compartments. Grey dashed lines indicate the borders of the oral epithelium. (**D,E**) Quantification of epithelial surface area in anteroposterior and lingual/labial regions of E13 (**D**), E13.5 (**E**), and E14 (**F**) incisor germs. (**G–I**) Quantification of total epithelial cell number in anterior–posterior and labial–lingual domains of E13 (**G**), E13.5 (**H**)**,** and E14 (**I**) incisors. Data are presented as mean ± s.d. (*n* = 8 per group). Anterior–posterior and labial–lingual measurements are paired; therefore, statistical significance was assessed using a two-sided Wilcoxon matched-pairs signed rank test. ns = *p* > 0.05, ** = *p* ≤ 0.01. Abbreviations: ant, anterior; df, dental furrow; dp, dental pulp; la, labial; li, lingual; oe, oral epithelium; post, posterior.

Lower incisor germs were oriented using anatomical features, and epithelial regions were defined by criteria generally used by most researchers in this field and according to previous publications [7,22,23]. At E13, when distinct labial and lingual domains could not yet be identified morphologically, we sampled the anterior and posterior halves of the epithelial bud. For comparisons across stages, these regions are labelled labial and lingual in the graphs, respectively, as anatomical reference labels only. They do not imply that the E13 regions are already distinct labial and lingual domains or establish lineage equivalence. At E13.5 and later, the compartments were delineated using the developing cervical loops, the positioning of the enamel knot [24,25,26,27,28], and the boundaries shown in Figure 1A,C. The same orientation and sampling criteria were applied across embryos and experiments.

### 2.3. Ex Vivo Explants Culture and Treatments

E12.5-E13 mandibles were dissected and cultured in 0.5% agarose-based semi-solid medium (Condalab, Madrid, Spain, #8010) supplemented with 20% foetal bovine serum (FBS; Gibco^®^, Grand Island, NY, USA, #A5256701), 1% penicillin-streptomycin (Gibco^®^, Grand Island, NY, USA, #15140148), and 50 mM ascorbic acid (Sigma-Aldrich, Saint Louis, MO, USA, #A92902) in Dulbecco’s modified eagle medium/nutrient mixture F-12 (DMEM/F12; Thermo-Fisher Scientific, Waltham, MA, USA, #10565018). For lineage tracing, at least ten E13 *Notch2-CreERT2/+;R26mT/mG* incisor explants were incubated with 100 nM 4-hydroxytamoxifen (4-OHT; Sigma-Aldrich, Saint Louis, MO, USA, #H7904) for 2 h. Following 4-OHT removal, separate samples were cultured for 24 and 48 h.

Cell-cycle length was measured in E13 wild-type lower incisors using 100 µM EdU dissolved in dimethyl sulfoxide (DMSO; Sigma-Aldrich, Saint Louis, MO, USA, #D4540) [29]. Five to eight separate E13 incisor explants were exposed to EdU for the indicated durations (1–24 h), with a distinct explant fixed in 4% PFA at each endpoint. Each explant therefore contributed to one time point only.

For Notch signalling inhibition, E12.5 *Keratin14-Cre/+;R26mT/mG* dissected mandibles were cultured in semi-solid medium containing 100 µM CB103 (MedChemExpress, New Jersey, NJ, USA, #HY-135145) or DMSO control for 48 h [30]. Five to eight independent incisors were evaluated per treatment and endpoint. Samples were fixed and processed for whole-mount immunofluorescence or RNA analysis.

For ex vivo organ cultures, only one lower incisor per embryo was used, and each explant was assigned to a single experimental condition and time point. Each incisor was considered an independent biological replicate and at least five independent biological replicates per condition and time point were included in the analyses. Explants were fixed and analysed at their respective endpoints; therefore, no repeated measurements were performed over time.

### 2.4. Tissue Preparation and Gene Expression Analysis

Whole embryos or dissected mandibles were collected in ice-cold phosphate-buffered saline (PBS; Gibco™, Thermo-Fischer Scientific, Waltham, MA, USA, #10-010-072) and fixed overnight at 4 °C in 4% paraformaldehyde (PFA; Sigma-Aldrich, Saint Louis, MO, USA, #P6148). For cryosectioning, samples were cryoprotected in 30% (w/v) sucrose (Sigma-Aldrich, Saint Louis, MO, USA, #S8501), embedded in Tissue-Tek OCT Compound (Sakura Finetek, Torrance, CA, USA, #4583), and sectioned at 10 µm using a Leica CM3050 S cryostat. For RNA analysis, E13–E14.5 explants were incubated with 2 U/mL Dispase II (Sigma-Aldrich, Saint Louis, MO, USA, #D4693-1G) in PBS for 20 min at room temperature to separate epithelium from mesenchyme. Epithelium was collected in 1.5 mL Eppendorf tubes containing TRIzol (Thermo-Fischer Scientific, Waltham, MA, USA, #15596026), homogenised with a motorised pestle, and sonicated for 30 min. cDNA synthesis was performed using the iScript™ cDNA Synthesis Kit (Bio-Rad, Basel, Switzerland, #1708891), and quantitative PCR was conducted with PowerTrack™ SYBR Green Master Mix (Thermo-Fisher Scientific, Waltham, MA, USA, #A46109) on an Illumina Eco™ system using 15 ng cDNA per reaction. For gene expression analysis with RT-qPCR, incisors from three to five embryos were pooled to generate each biological sample, with each pool originating from an independent litter. At least 5 biological replicates were used per stage, genotype, and condition.

### 2.5. Histology and Immunostaining

For *histology*, 10 µm cryosections of lower incisors were rehydrated in PBS and then stained with haematoxylin (Merck, Darmstadt, Germany, #109249) and eosin (Sigma-Aldrich, Saint Louis, MO, USA, #230251-25G).

For *immunohistochemistry*, rehydrated cryosections of lower incisors were permeabilised with 0.1% Triton X-100 (Sigma-Aldrich, Saint Louis, MO, USA, #T8787) in PBS for 15 min, and antigen retrieval was performed in 10 mM citrate buffer (pH 6.0) at 80 °C for 30 min. Endogenous peroxidase was quenched with 1% H_2_O_2_ in methanol for 10 min. Sections were blocked with 1% bovine serum albumin (BSA; Carl Roth GmbH, Mannheim, Germany, #0163.2), and 10% goat serum (Gibco, Grand Island, NY, USA, #2426173) for 30 min, incubated with primary antibodies (1:100) overnight at 4 °C, followed by biotinylated anti-rabbit secondary antibody (1:250) for 1 h. Signal was detected using the Vectastain^®^ ABC-HRP Kit (Vector Laboratories, Newark, CA, USA, #PK-4001 for rabbit IgG, #PK-6105 for goat IgG) and developed with the AEC Substrate Kit (Vector Laboratories, Newark, CA, USA, #SK4200). Sections were counterstained with toluidine blue (Sigma-Aldrich, Saint Louis, MO, USA, #T3260) and mounted in Glycergel (Agilent Dako, Santa Clara, CA, USA, #C0563).

For *immunofluorescence*, cryosections of lower incisors were blocked with 10% donkey serum (BioWest, Nuaillé, France, #S2170-100) and 1% BSA for 30 min, incubated with primary antibodies overnight at 4 °C, followed by secondary antibodies (1:500) and DAPI (1:1000; Tocris Bioscience, Bristol, UK, #5748), and finally mounted with a fluorescence mounting medium (Agilent Dako, Santa Clara, CA, USA, #S3023). DNA-incorporated EdU was detected using the Click-iT^®^ EdU Imaging Kit (Life Technologies, Carlsbad, CA, USA, #C10640). Primary polyclonal antibodies included Notch2 (Cell Signaling, Danvers, MA, USA, #5732; 1:100), Green Fluorescent Protein (GFP; Abcam, Cambridge, UK, #ab6673; 1:100), Ki67 (Abcam, Cambridge, UK, #ab15580; 1:100), Phosphohistone-3 (PH3; Cell Signaling, Cambridge, UK, #3377; 1:100), and BrdU (Abcam, Cambridge, UK, #ab6326; 1:100).

For *whole mount immunofluorescence*, E13–E14 mandibles were fixed in 4% PFA overnight at 4 °C, permeabilized in 1% Triton X-100 for 24 h, and blocked in 1% BSA and 10% donkey serum for 24 h at 4 °C. EdU detection was performed using a freshly prepared Click-iT^®^ EdU solution for 72 h at 4 °C. Samples were cleared in 88% Histodenz (Sigma-Aldrich, Saint Louis, MO, USA, #D2158-100G) in PBS and imaged on a Leica Stellaris 5 confocal microscope using a 20x dry objective (HC PL APO CS2, NA 0.75). Z-stack images were merged in LAS X version 4.5 software.

### 2.6. Laser Capture Microdissection and RNA Sequencing

In total, eighty E13-E13.5 wild-type and mutant embryos were fixed in 4% PFA, dehydrated in ethanol, cleared in xylene, and paraffin-embedded. Sections of lower incisors 10 µm thick were placed on PEN membrane glass slides (Applied Biosystems, Foster City, CA, USA, #LCM0522), deparaffinised, rehydrated, and stained with 0.05% Cresyl Violet (Merck, Darmstadt, Germany, #C5042) in 100% ethanol. Target tissues were dissected using the laser microdissection ArcturusXT system (Thermo-Fischer, Waltham, MA, USA) and collected in CapSure™ Macro LCM Caps (Applied Biosystems, Foster City, CA, USA, #LCM0211). Samples were sonicated for 30 min, and RNA libraries were prepared using Takara Clontech PicoMammalian v2 protocol. Both lower incisors from five embryos were pooled per stage, epithelial compartment, and genotype to generate each biological sample. The analysis included 4 independent biological samples per condition. Sequencing was performed at the Functional Genomics Centre Zurich on a NovaSeq X Plus (Illumina, Inc, San Diego, CA, USA) with 150 bp paired-end reads, generating 24–39 million reads per sample. Bulk RNA-seq analysis was performed using the SUSHI framework and included quality assessment with FastQC, adapter trimming with fastp, pseudo-alignment and quantification using Kallisto Bioconductor R package with GENCODE GRCm39 (Release M31) reference, and differential expression with DESeq2 [31,32,33,34]. Gene ontology (GO) pathway analysis was performed with clusterProfiler Bioconductor R package using “enrichGO” and “gseGO” functions in R 4.4.0 with Bioconductor 3.19. Figures were generated using the exploreDE Shiny application [35,36,37].

### 2.7. Image Analysis

Serial cryosections (10 µm) of E13–E14 incisors were labelled with DAPI, Ki67, PH3, EdU, and BrdU. Labial and lingual epithelia were segmented in ImageJ 1.54p with training data generated in Ilastik, following the orientation and regional definition guidelines mentioned above [14,15]. Segmentation models were imported into Imaris (Bitplane; https://imaris.oxinst.com/packages, accessed on 1 June 2024) for 3D cell segmentation and nuclei counting. Epithelial area was quantified in ImageJ. Cumulative EdU assays were used to estimate cell cycle length (Tc) minus the S-phase duration (Ts) [29]. For immunostaining and image-based quantifications, including Ki67, EdU, BrdU, and PH3 analyses, at least eight embryos were analysed per developmental stage (E13, E13.5, E14, and E14.5) with only one incisor used per embryo. Labial and lingual measurements were obtained from the same incisor and therefore considered paired observations. For each incisor, multiple sections spanning its entire length were analysed to account for variations arising from its three-dimensional structure. Measurements from these sections were combined and averaged to obtain a single value per epithelial compartment and incisor. Individual sections or images were not considered independent biological replicates.

### 2.8. Quantification and Statistical Analysis of Biological Replicates

Quantitative data were analysed using GraphPad Prism version 10.6.1. Statistical tests were selected according to the experimental design, including the number of independent groups, paired or unpaired measurements, and the factors under investigation. Depending on the experiment, statistical significance was assessed using unpaired t-tests, two-way analysis of variance (ANOVA), repeated-measures two-way ANOVA, or mixed-effects analysis, followed by the appropriate multiple-comparisons correction (Šídák’s or Holm–Šídák’s test). For RT-qPCR experiments, statistical analyses were performed on ΔCt values, while relative expression levels are presented as fold changes. For statistical significance: ns = *p* > 0.05; * = *p* ≤ 0.05; ** = *p* ≤ 0.01; *** = *p* ≤ 0.001; **** = *p* ≤ 0.0001.

## 3. Results

### 3.1. Epithelial Labial-Lingual Asymmetry Emerges Early in Incisor Development

To characterise the onset of asymmetry, we performed whole-mount imaging of embryonic days 13 to 14 (E13–E14) *Keratin14Cre/+;R26mT/mG* incisors, in which the developing epithelium is labelled with green fluorescent protein (GFP) (Figure 1A,B). At E13, the epithelium formed a symmetrical bud. By E13.5, the first morphological signs of labial–lingual asymmetry appeared, and by E14, labial and lingual cervical loops were clearly distinct (Figure 1B). Quantitative analysis of epithelial area and total cell number confirmed that labial growth increased significantly between E13.5 and E14, while lingual parameters remained largely unchanged (Figure 1C–E). These findings indicate that directed epithelial expansion initiates early and is labial-specific.

### 3.2. Differential Cell-Cycle Kinetics Drive Epithelial Asymmetry

**Figure 2:**
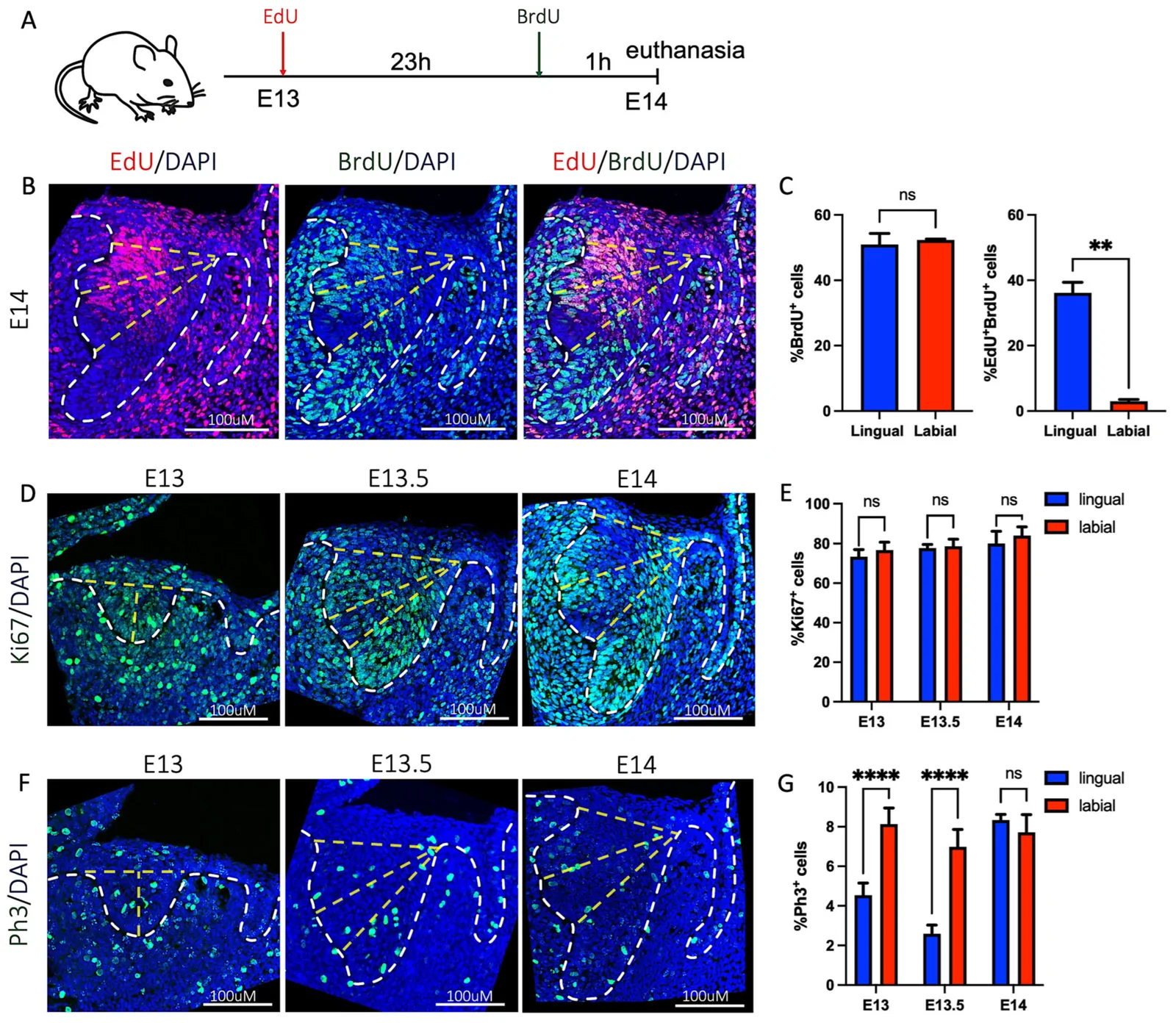
Temporal and regional variations in cell-cycle dynamics during asymmetric incisor development. (**A**) Experimental design for dual labelling of proliferating cells. Pregnant females were injected with EdU at E13 and with BrdU 1 h before euthanasia at E14. (**B**) Representative fluorescent images showing EdU, BrdU, and merged staining in E14 incisors. (**C**) Quantification of BrdU-positive cells and double EdU/BrdU-positive cells in lingual and labial epithelia of E14 incisors. Data are presented as mean ± s.d. (*n* = 3 per group). Statistical significance was assessed using a two-sided Wilcoxon matched-pairs signed-rank test. ns = *p* > 0.05, ** = *p* ≤ 0.01. (**D**) Immunofluorescence for Ki67 in E13–E14 incisors. (**E**) Quantification of Ki67-positive cells in lingual and labial epithelia of E13–E14 incisors. (**F**) Immunofluorescent staining for phospho-histone H3 (PH3) in E13–E14 incisors. (**G**) Quantification of PH3-positive cells in lingual and labial epithelia. Nuclei are labelled with DAPI. White dashed lines outline the incisor epithelium. Yellow dashed lines define the boundary between the compartments that were used for quantification. Data are presented as mean ± s.d. (*n* = 5 per group). Statistical significance in (**D**–**G**) was assessed using two-way ANOVA followed by Šídák’s multiple comparisons test. ns = *p* > 0.05, ** = *p* ≤ 0.01, **** = *p* ≤ 0.0001.

To investigate the cellular basis of asymmetry, we performed dual EdU/BrdU pulse-chase labelling (Figure 2A). A short BrdU pulse labelled a similar fraction of cells in both labial and lingual epithelia, indicating that the initial cycling population was comparable in the two areas (Figure 2B,C), corroborated by Ki67 immunostaining (Figure 2D,E). However, after 24 h, only a few EdU-labelled cells were observed in the labial epithelium, whereas most lingual cells had retained the EdU label, indicating fewer cell-cycle entries in the lingual epithelium (Figure 2B,C). Phospho-histone H3 (PH3) staining revealed higher mitotic activity in labial cells (Figure 2F,G). Together, these findings indicate that regional differences in cell-cycle kinetics, rather than the number of proliferating cells, drive the onset of epithelial asymmetry.

**Figure 3:**
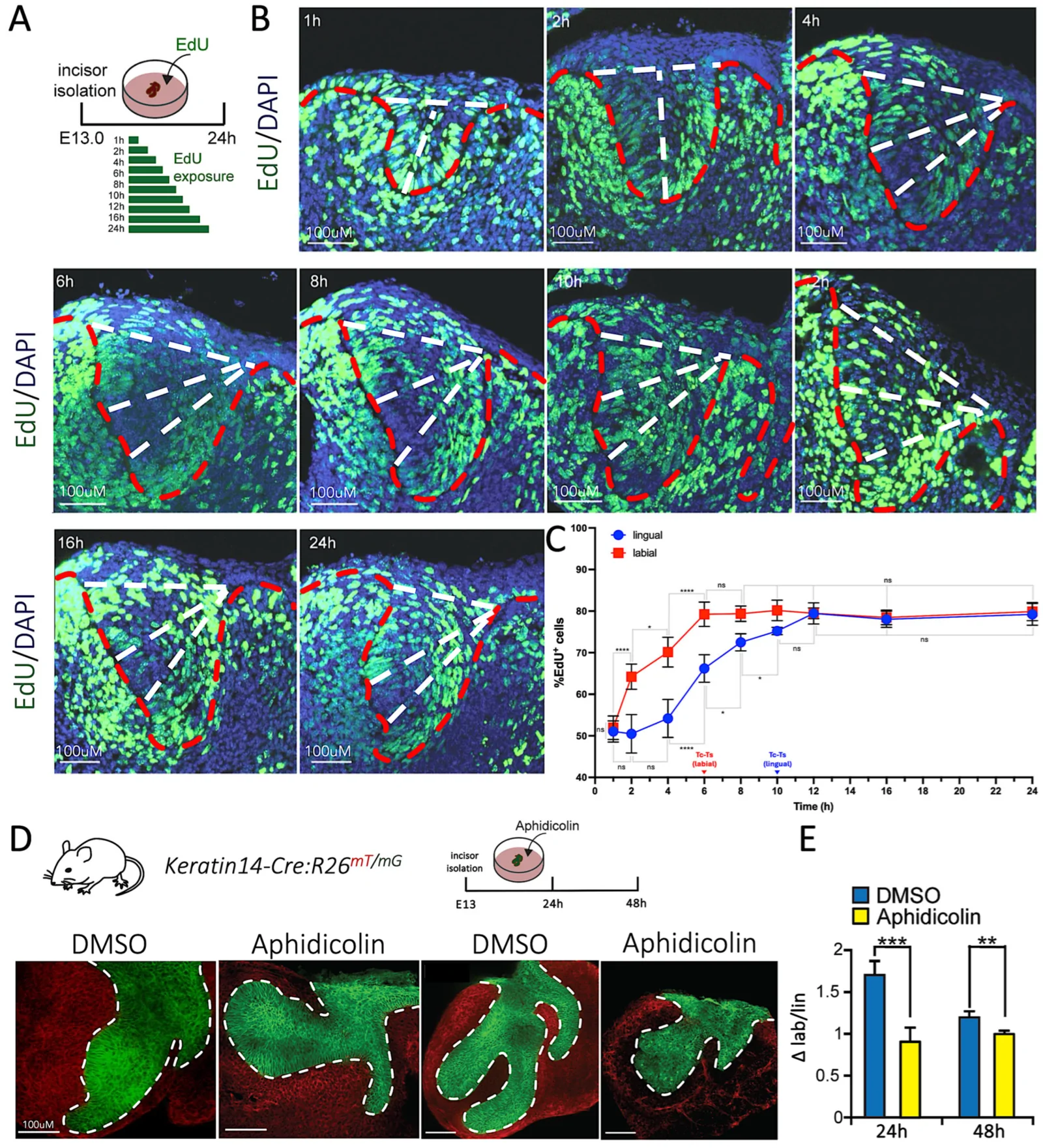
Differential cell-cycle duration underlies incisor epithelium asymmetry. (**A**) Schematic of the EdU cumulative-labelling experiment. (**B**) Representative fluorescent images of E13 incisor explants incubated with EdU (green). Nuclei are labelled with DAPI (blue). Red dashed lines indicate the outline of the incisor epithelium, while white dashed lines show the areas that were sampled for counting. (**C**) Quantification of EdU-positive cells over time in lingual and labial epithelia. Data represent mean ± s.d. Per time point *n* = 5–8 embryos, with every embryo contributing only 1 incisor. Statistical significance was assessed using two-way ANOVA with repeated measures for the paired labial and lingual compartments, followed by Šídák’s multiple-comparisons test. For simplicity, only the significance between different time points is shown in the graphs. (**D**) Ex vivo culture of E13 *Keratin14Cre/+;R26mT/mG* incisors treated with DMSO (control) or the cell-cycle inhibitor aphidicolin for 24 or 48 h. (**E**) Quantification of the labial-to-lingual epithelial ratio in DMSO- and aphidicolin-treated incisors at 24 and 48 h. Data are presented as mean ± s.d. (*n* = 5 per group). Statistical significance was assessed using two-way ANOVA followed by Šídák’s multiple comparisons test. ns = *p* > 0.05, * = *p* ≤ 0.05, ** = *p* ≤ 0.01, *** = *p* ≤ 0.001, **** = *p* ≤ 0.0001.

Cumulative EdU labelling on E13 incisor explants also revealed different labelling kinetics in the labial and lingual epithelium. Ex vivo cultures were exposed to EdU for 1–24 h (Figure 3A). After 1 h, 52 ± 2.8% of labial and 51 ± 2.6% of lingual cells were labelled (Figure 3B). By 2 h, 64.2 ± 3% of labial cells were EdU-positive. Labelling increased significantly and gradually to 79.4 ± 2.9% by 6 h and plateaued (Figure 3C). In contrast, EdU incorporation in the lingual epithelium remained unchanged until 4 h, then rose to 79.4 ± 2.5% by 10 h and plateaued (Figure 3C). Assuming that cells divide at a relatively constant rate and that the plateau represents the total proliferating population, the time required to reach the plateau corresponds to the cell-cycle duration (Tc) minus the S-phase duration (Ts) [29,38]. Thus, the earlier saturation observed in the labial epithelium is consistent with faster cell-cycle progression compared to the lingual compartment. The plateau fraction of EdU-labelled cells in both epithelia closely matched the proportion of Ki67-positive cells observed in vivo (Figure 2D,E). Explants treated with the DNA replication inhibitor aphidicolin for 24 to 48 h remained at the bud stage, whereas DMSO-treated controls progressed towards the cap stage (Figure 3D,E), showing that DNA replication is required for normal developmental progression under these culture conditions. This experiment does not isolate a specific role for proliferation in establishing asymmetry.

### 3.3. Transcriptional Changes Accompany the Establishment of Labial-Lingual Asymmetry

**Figure 4:**
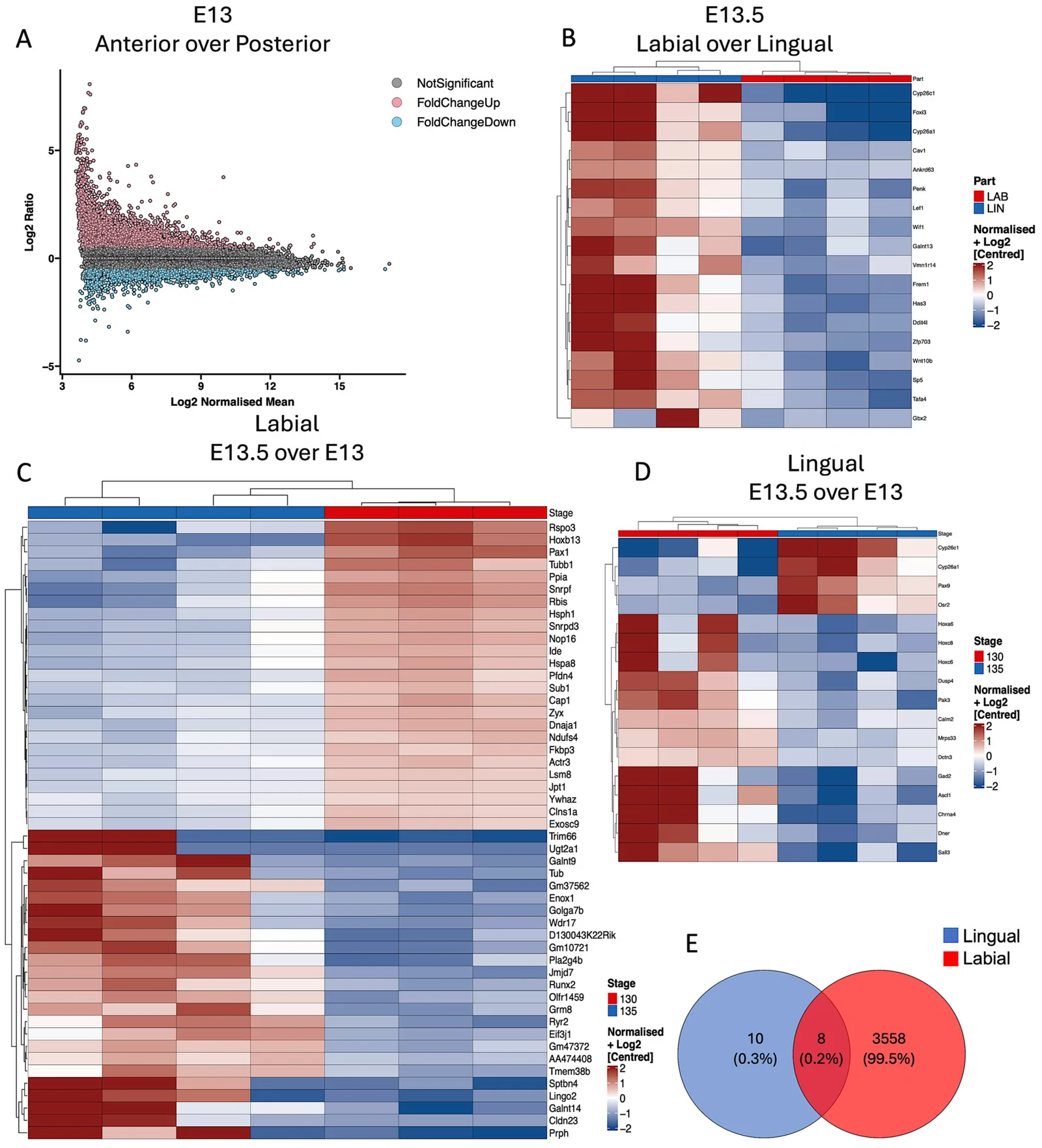
Transcriptomic analysis reveals the emergence of distinct labial and lingual epithelial expression profiles during early incisor development. (**A**) Scatter plot showing differential gene expression between the anterior and posterior epithelial regions of E13 wild-type incisor germs. The x-axis represents log_2_ normalised mean (anterior versus posterior), and the y-axis represents the log_2_ ratio. (**B**) Heatmap of 18 differentially expressed genes between the labial and lingual epithelial compartments at E13.5. (**C**) Heatmap of 449 genes significantly upregulated in the labial epithelium during the transition from E13 to E13.5. (**D**) Heatmap of 17 genes significantly upregulated in the lingual epithelium from E13 to E13.5. Columns represent independent biological samples, and rows represent genes. Colours indicate centred, log_2_-transformed normalised expression values. (**E**) Venn diagram showing 8 shared genes out of 3576 total genes expressed in both epithelia between E13 and E13.5.

To assess the molecular underpinning of these differential dynamics, we performed bulk RNA sequencing of laser-captured lingual and labial epithelia from E13-E13.5 incisor germs. At E13, comparison of the anterior and posterior epithelial regions revealed no statistically significant differentially expressed genes (DEGs) (Figure 4A). By E13.5, transcriptomic analysis identified 18 DEGs between the labial and lingual epithelial compartments. Among these, Foxi3, Lef1, and Wnt10b showed higher expression in the lingual epithelium (Figure 4B). During the E13 to E13.5 transition, 449 genes changed expression in the labial compartment and 17 in the lingual compartment (Figure 4C,D). Eight differentially expressed genes were shared between the two developmental comparisons (Figure 4E), indicating that most detected changes were compartment specific.

### 3.4. Labial-Specific Cell-Cycle Dynamics and Notch2 Lineage Restriction Drive Epithelial Asymmetry

**Figure 5:**
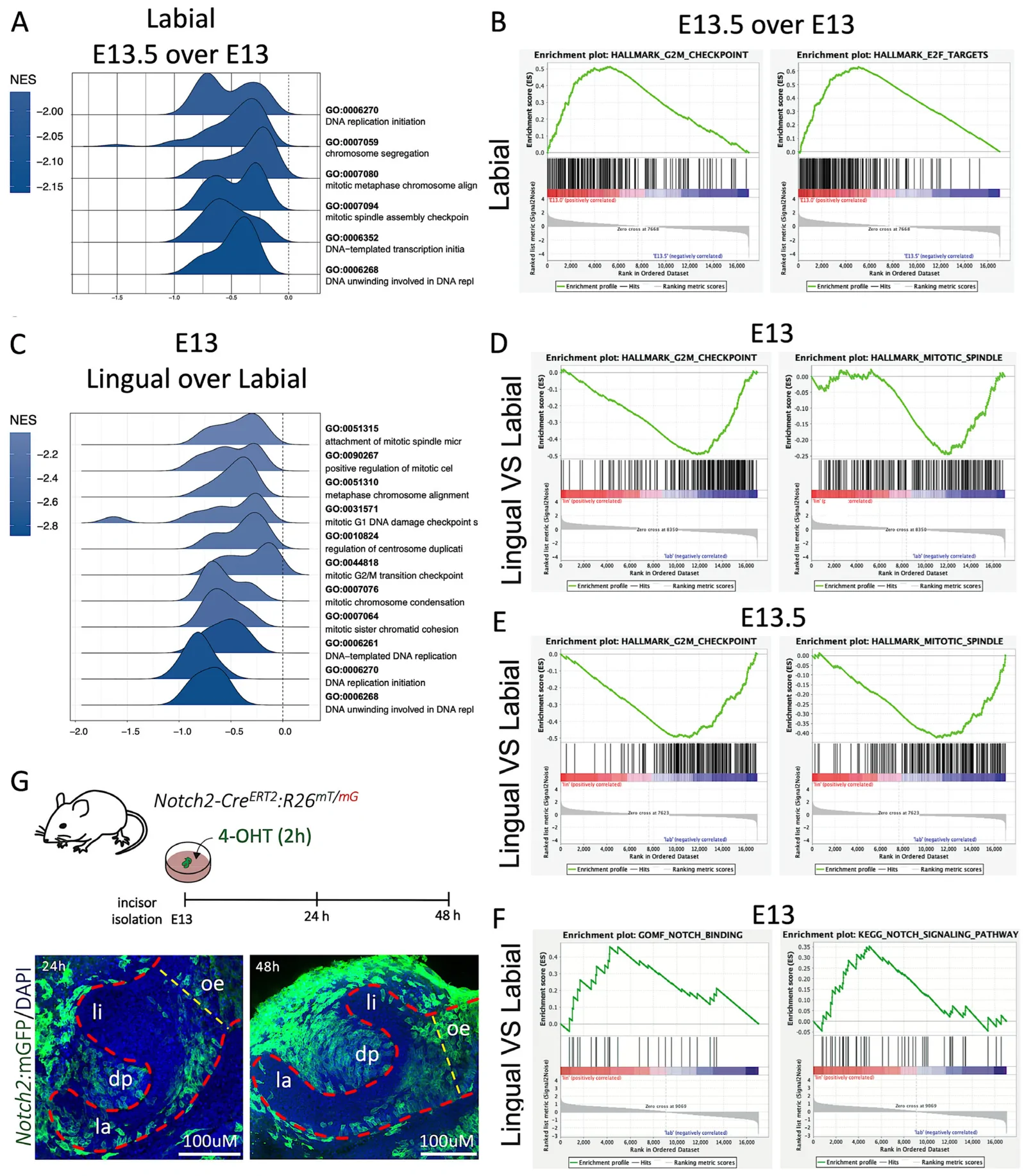
Transcriptomic analysis of lingual and labial epithelia reveals fine regulation of proliferation and Notch signalling prior to asymmetry generation. (**A**) Ridge plot showing the distribution of cell-cycle-related genes in labial epithelium at E13.5 versus E13. (**B**) Gene set enrichment analysis (GSEA) indicating enrichment of G2/M checkpoint and E2F-target genes in E13 labial epithelium. (**C**) Ridge plot comparing cell-cycle-related gene enrichment between lingual and labial epithelia at E13. (**D**,**E**) GSEA comparing lingual versus labial epithelia at E13 (**D**) and E13.5 (**E**), showing higher enrichment of G2/M checkpoint and mitotic spindle genes in labial epithelium. (**F**) GSEA comparing labial and lingual epithelia at E13, showing enrichment of Notch signalling components in labial epithelium. Differential expression analysis was performed using DESeq2 with Benjamini–Hochberg correction; *p* < 0.05 and |log_2_ fold change| > 1. (**G**) Whole-mount Z-stack imaging of *Notch2-CreERT2;R26mT/mG* E13 incisor germs after 24 h and 48 h in ex vivo culture. Red dashed lines indicate the borders of the incisor epithelium and yellow dashed lines indicate the borders of the oral epithelium.

To further investigate the molecular mechanisms underlying differential epithelial growth, we examined the developmental regulation of proliferation-associated pathways in the labial and lingual compartments. Ridge plot analysis of labial differentially expressed genes revealed a pronounced shift in those associated with DNA replication and cell-cycle progression between E13 and E13.5 (Figure 5A). Gene-set enrichment analysis showed that at E13, the labial epithelium is enriched for pathways regulating the G2/M transition and S-phase progression, indicative of tight transcriptional control of cell-cycle dynamics (Figure 5B). Comparative analysis confirmed stronger enrichment in labial versus lingual epithelia for mitotic spindle formation and G2/M transition pathways across E13-E13.5 (Figure 5C–E). These results show that cell-cycle-related gene sets were enriched in the labial epithelium in the comparisons described above. Together with the cell-labelling data, this pattern is consistent with regional differences in cell-cycle dynamics before and during epithelial asymmetry. Gene-set enrichment also highlighted Notch signalling among labial-specific pathways (Figure 5F) but does not demonstrate by itself that Notch directly coordinates epithelial cell proliferation and fate specification prior to incisors rotation.

Previous studies have revealed that Notch2 is restricted to the labial epithelium at E13 and E13.5 [7]. To assess the contribution of Notch2-expressing cells and their progenitors during the bud-to-cap transition, we performed lineage tracing using *Notch2-CreERT2/+;R26mT/mG* incisor germs. E13 incisors were cultured ex vivo with 4-Hydroxytamoxifen (4-OHT) for 2 h to induce GFP expression, followed by semisolid culture for 24 or 48 h. At 24 h, Notch2-expressing cells and their progeny were predominantly localised to the basal and suprabasal layers of the labial epithelium (Figure 5G). By 48 h, GFP-positive cells remained confined to the basal and suprabasal epithelial layers of the developing labial cervical loop (Figure 5G). This pattern is consistent with the developmental changes in Notch2 expression observed in tissue sections, where Notch2 is predominantly labial at E13.5 but extends towards the lingual compartment by E14.5, confirmed also with immunohistochemistry (Figure S1). These data indicate that Notch2-expressing cells mark and contribute to the expanding labial domain, reinforcing epithelial asymmetry during incisor development.

### 3.5. Epithelial Notch2 Deletion Alters the Expression of Notch Pathway Components 

**Figure 6:**
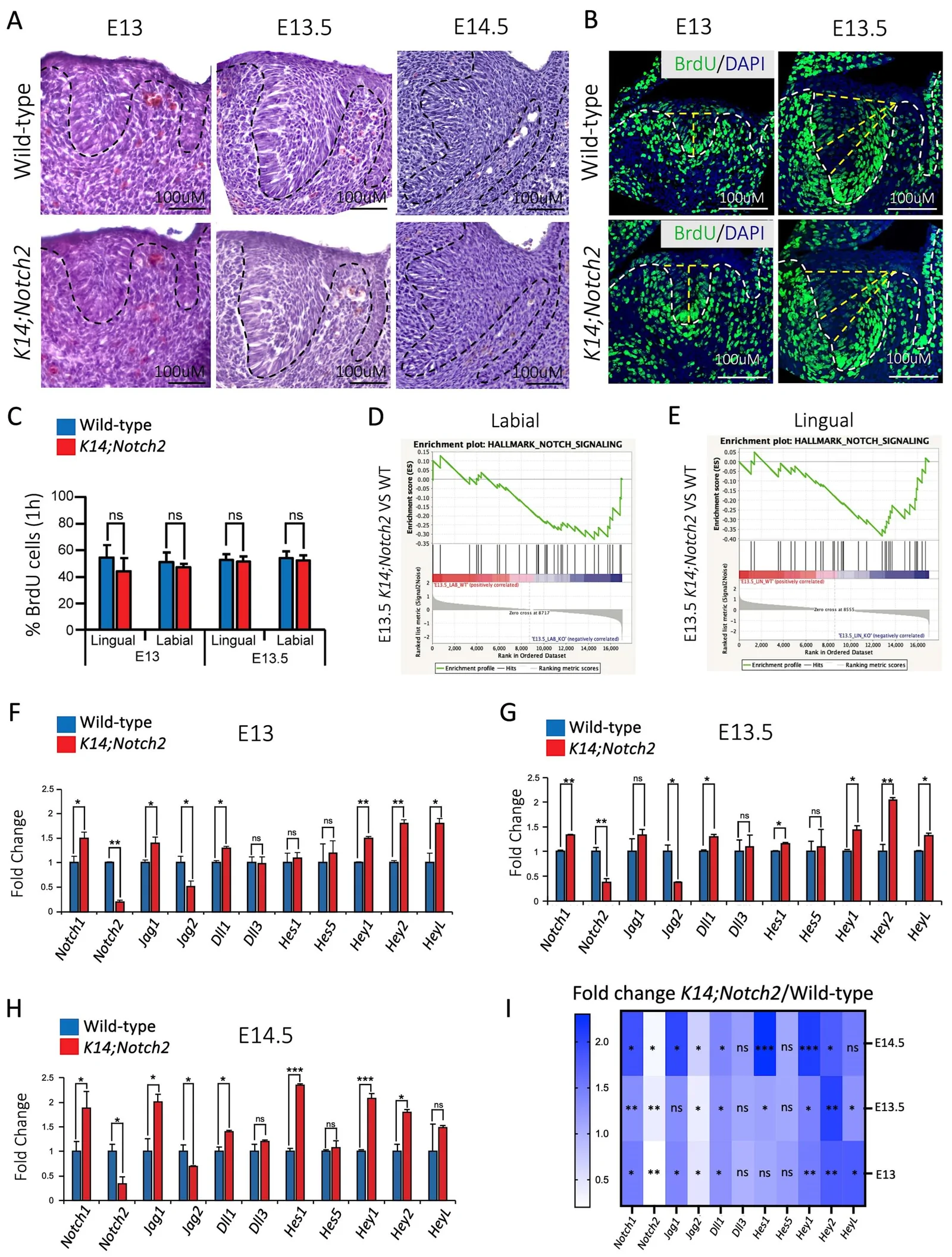
Notch2 deletion in the incisor epithelium triggers compensatory mechanisms. (**A**) Histology of E13–E14.5 wild-type and *Keratin14Cre/+;Notch2fl/fl* (*K14;Notch2*) incisors. (**B**) Immunofluorescence for BrdU (green) in E13-E13.5 wild-type and *K14;Notch2* incisors. Pregnant females were injected with BrdU 1 h before euthanasia. Nuclei are labelled with DAPI (blue). White dashed lines indicate the boundaries of the incisor germs while the yellow dashed lines indicate the areas that were sampled for counting. (**C**) Quantification of BrdU-positive cells in lingual and labial epithelia. Data are presented as mean ± s.d. Statistical significance was assessed using two-way ANOVA with repeated measures for epithelial compartment, followed by Šídák’s multiple-comparisons test. ns = *p* > 0.05. (**D**,**E**) GSEA of Notch signalling-related genes in lingual and labial epithelia of *K14;Notch2* incisors at E13 (**D**) and E13.5 (**E**). (**F**–**H**) Quantitative RT-PCR of Notch pathway components in wild-type and *K14;Notch2* epithelia at E13 (**F**), E13.5 (**G**) and E14.5 (**H**), presented as fold change relative to wild-type. Statistical analyses were performed on ΔCt values using multiple unpaired t-tests with Holm–Šídák correction for multiple comparisons within each developmental stage. Data are presented as mean ± s.d. (*n* = 5 biological replicates per stage and genotype). (**I**) Heatmap summarising RT-qPCR analysis of Notch pathway components in *K14;Notch2* incisors relative to wild-type controls at E13, E13.5, and E14.5. Each column represents a gene, and each row corresponds to a developmental stage. Colour intensity reflects the relative fold change in gene expression, whereas statistical significance is indicated by the corresponding symbols. ns = *p* > 0.05, * = *p* < 0.05, ** = *p* ≤ 0.01, *** = *p* ≤ 0.001.

To investigate the function of Notch2 in incisor epithelial development, we analysed *Keratin14Cre/+;Notch2fl/fl* (*K14;Notch2*) mutants alongside wild-type controls. No obvious morphological differences were detected in the Notch2 conditional mutants at E13–E14.5 (Figure 6A). Similarly, cell proliferation was not affected at E13 and E13.5 (Figure 6B,C). These findings do not exclude effects at later stages. The observed upregulation of other Notch pathway components is consistent with possible compensatory mechanisms but does not establish that compensation caused the absence of an early phenotype.

To investigate molecular consequences of *Notch2* loss, we performed bulk RNA sequencing of labial and lingual epithelia from wild-type and mutant incisor germs. Gene set enrichment analysis revealed overlapping peaks in Notch signalling regulation, prompting further quantitative assessment (Figure 6D,E). Quantitative RT-PCR on *K14;Notch2* and wild-type epithelia, analysed before and after asymmetry establishment, demonstrated a significant upregulation of *Notch1* in the mutant epithelium (Figure 6F–H). Additionally, *Jag1*, *Dll1*, and canonical Notch targets (*Hes1*, *HeyL*, *Hey1*, and *Hey2*) were upregulated, whereas *Jag2* was reduced and *Hes5* remained unchanged (Figure 6F–H). Together, these findings suggest that a possible compensatory activation of additional Notch receptors or crosstalk with other signalling pathways may buffer the effects of Notch perturbation during early epithelial asymmetry.

### 3.6. Pharmacological Inhibition of Notch Signalling Disrupts Proliferation Asymmetry

**Figure 7:**
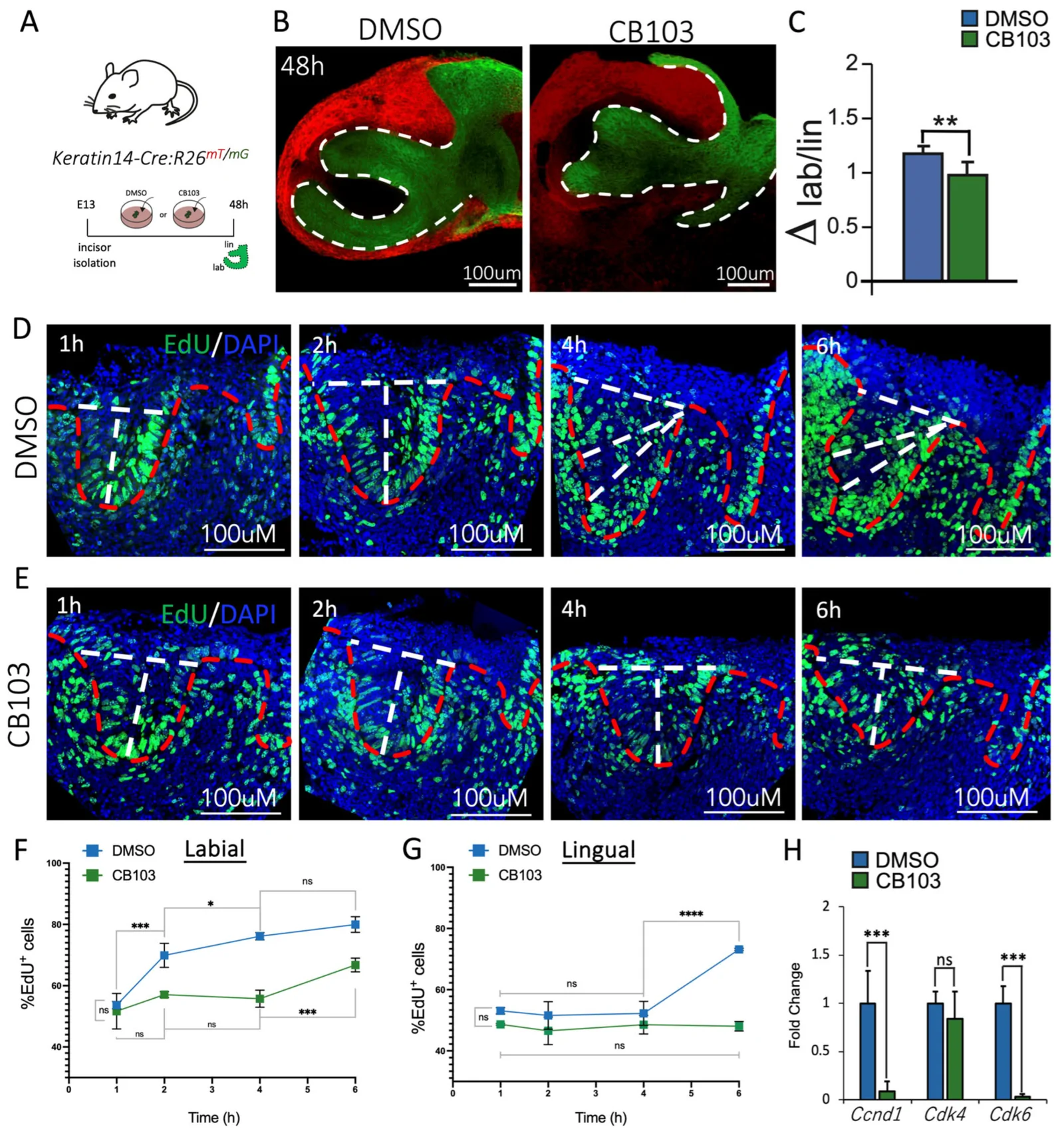
Pharmacological inhibition of Notch signalling alters cell-cycle progression of incisor explants. (**A**) Schematic representation of incisor organotypic cultures in semi-solid medium, treated for 2 days with DMSO (control) or the pan-Notch inhibitor CB103. (**B**) Whole-mount imaging of E13 *Keratin14-Cre*/+*;R26mT/mG* incisor organotypic cultures. (**C**) Quantification of the labial-to-lingual epithelial area ratio in DMSO- and CB103-treated incisors. Red dashed lines outline the incisor epithelium, while white dashed lines indicate the areas that were used for counting. Data are presented as mean ± s.d. For each condition and time point, eight independent incisor germs were used for analysis. Statistical significance was assessed using ordinary two-way ANOVA followed by Šídák’s multiple-comparisons test. (**D**,**E**) Representative immunofluorescence images of EdU incorporation (green) in DMSO-treated (**D**) and CB103-treated (**E**) explants. E12.5 incisors were pre-cultured for 12 h with DMSO or CB103 before EdU addition. Explants were collected at multiple intervals for imaging and RNA extraction. Nuclei are labelled with DAPI (blue). Red lines indicate the borders of the incisor epithelium and the epithelial compartments that were used for counting. (**F**,**G**) Quantification of cumulative EdU incorporation in the labial (**F**) and lingual (**G**) epithelial compartments of developing incisor explants cultured with DMSO (control) or the Notch inhibitor CB103. Explants were exposed to EdU for 1, 2, 4, or 6 h, and the percentage of EdU-positive cells was quantified relative to the total number of DAPI-positive cells in the corresponding compartment. DMSO- and CB103-treated incisors represent independent samples, and separate incisors were analysed at each time point. Data are presented as mean ± s.d. Statistical significance was assessed using two-way ANOVA of paired labial–lingual differences with treatment and time as factors. For simplicity, only the significance between different time points is shown in the graphs. (**H**) Quantitative RT-PCR analysis of the cell-cycle regulators *Ccnd1*, *Cdk4*, and *Cdk6* in DMSO- (blue) and CB103-treated (green) epithelia. Data are presented as mean ± sd (*n* = 5 per condition). Statistical significance was assessed using multiple unpaired t-tests on ΔCt values with Holm–Šídák correction. ns = *p* > 0.05, * = *p* ≤ 0.05, ** = *p* ≤ 0.01, *** = *p* ≤ 0.001, **** = *p* ≤ 0.0001.

To circumvent potential compensatory mechanisms, E13 *Keratin14Cre/+;R26mT/mG* incisor germs were cultured ex vivo and treated with the Notch inhibitor CB103 or DMSO as a control (Figure 7A). CB103-treated explants did not exhibit the characteristic asymmetric epithelial growth observed in controls (Figure 7B), and the labial-lingual area difference was significantly reduced after 48 h (Figure 7B,C).

To assess effects on cell cycle progression, E12.5 explants were cultured for 12 h with CB103 or DMSO, followed by cumulative EdU labelling for 1–6 h (Figure 7D,E). After 1 h, 49 ± 1.7% of lingual and 49 ± 5.4% of labial cells in CB103-treated explants were EdU-positive, comparable to controls (52.9 ± 4.3% lingual and 53.1 ± 2.3% labial) (Figure 7F,G), indicating that the initial proportion of labelled cells was unaffected. Cumulative EdU incorporation in the labial side increased progressively in control explants (Figure 7F). However, CB103-treated labial cells showed a delayed increase in EdU incorporation, with relatively stable labelling between 2 and 4 h, followed by a significant increase to 68 ± 3.8% at 6 h (Figure 7F). In the lingual epithelium, EdU incorporation increased in control explants but remained stable following CB103 treatment (Figure 7G). These findings show that CB103 treatment altered cumulative EdU incorporation in both epithelial compartments over the measured time course, with distinct temporal responses in the labial and lingual compartments.

qRT-PCR for key S-phase regulators showed that CB103 significantly reduced *Ccnd1* and *Cdk6*, whereas *Cdk4* remained unchanged (Figure 7H). These results indicate that Notch signalling modulates cell-cycle progression in the labial epithelium, potentially through *Ccnd1* and *Cdk6*, suggesting a possible mechanistic link between pathway activity, cell-cycle kinetics and directional epithelial growth.

## 4. Discussion

Cell proliferation is a fundamental driver of morphogenesis and organ shaping, including during tooth development [39]. Rodent incisors provide a powerful model for investigating how differential growth generates asymmetry in epithelial appendages [2,39]. Our data demonstrate that directional incisor growth arises not from differences in the proportion of proliferating cells, but from regional variation in cell-cycle kinetics. Labial epithelial cells progress through the cell cycle approximately twice as fast as their lingual counterparts, driving preferential labial expansion and epithelial rotation during the bud-to-cap transition. Notably, the cell-cycle length of the labial epithelial cells is comparable to that of other rapidly proliferating populations, including embryonic stem cells during germ layer formation and intestinal progenitors [40,41]. Consistent with these findings, transcriptomic profiling revealed enrichment for genes involved in DNA replication, G2/M progression, and mitotic spindle assembly within the labial epithelium. Together, these results indicate that modulation of cell-cycle timing, rather than changes in the overall number of proliferating cells, is a key determinant of tissue growth dynamics and the emergence of epithelial asymmetry.

A recent study demonstrated that both the number of cells and the spatial extent of the labial epithelium of the incisor bud increase between E13 and E13.5, before stabilising by E14.5 [42]. However, these conclusions were based solely on analyses of proliferative activity in a single epithelial cell population (i.e., the basal epithelial cells), while other epithelial cell populations were not examined. In the present study, we quantified cell-cycle dynamics across the entire dental epithelial compartment, including cells of the inner bud epithelium. Consistent with Ki67 staining, our analyses revealed that most dental epithelial cells are actively cycling during the bud-to-cap transition. Our findings demonstrate that differential growth of the incisor epithelium is driven by changes in cell-cycle kinetics throughout the dental epithelium, rather than by proliferation restricted to the basal epithelial cell population. This raises the question of which signalling pathways regulate these growth dynamics and thereby establish epithelial asymmetry during incisor morphogenesis.

Transcriptomic analyses suggest that the emergence of morphological asymmetry coincides with the development of distinct gene expression profiles in different epithelial regions. Transcriptional differences were detected between the apparent labial and lingual compartments of the developing incisor. Several regionally expressed genes, including *Lef1*, *Foxi3*, and *Wnt10b*, are linked to epithelial development and odontogenesis [25,26,27,28]. *Foxi3* contributes to ectodermal appendage and tooth crown development, while Wnt signalling is essential for dental epithelial patterning and differentiation [25,27,28]. Previous studies have also shown interactions between Wnt-associated transcriptional regulators, including LEF1 and PITX2 during incisor development [26]. In the lingual epithelium, expression of several *Hox* genes, including *Hoxa6*, *Hoxc6*, and *Hoxc8*, also changed during development. These genes are associated with anterior–posterior patterning and regional identity in the embryo [43]. Together, these findings suggest that the establishment of labial–lingual asymmetry involves changes in gene expression related not only to cell-cycle regulation, but also to epithelial identity and developmental patterning.

Our data identify Notch signalling as a key regulator of this process. Notch2 is asymmetrically distributed within the incisor epithelium before epithelial rotation, consistent with previous reports [6,7]. Lineage tracing further demonstrates that *Notch2*-expressing cells and their progenitors preferentially contribute to the labial domain. Together with the enrichment of canonical Notch target genes, these findings link spatially restricted *Notch2* activity to differential epithelial proliferation.

Functional experiments support this model. Pharmacological inhibition of Notch signalling does not prevent epithelial invagination or cervical loop formation but instead equalises growth between the labial and lingual epithelia, resulting in a more molar-like morphology. Our findings suggest that Notch signalling contributes to the regulation of cell-cycle progression. Consistent with this role, pharmacological Notch inhibition significantly downregulated the G1/S phase regulators *Ccnd1* and *Cdk6* [44,45]. These findings establish a possible link between localised Notch activity and cell-cycle control, providing a mechanism by which regional differences in proliferation may generate epithelial asymmetry.

By contrast, a recent study implicated asymmetric Shh expression in early incisor morphogenesis and reported that genetic or pharmacological inhibition of Shh signalling disrupts epithelial invagination and impairs the acquisition of asymmetry [42]. However, because Shh inhibition arrests the bud-to-cap transition itself, it is difficult to distinguish specific effects on epithelial asymmetry from broader defects in tooth morphogenesis. The more selective phenotype produced by Notch inhibition therefore provides stronger evidence that differential epithelial growth is a primary driver of incisor asymmetry.

The present study does not show how Notch and Shh signalling relate to each other during early mouse incisor development. Our experiments and those in the previous study [42] examined different aspects of development, so they cannot establish whether one pathway controls the other, whether the pathways interact directly, or whether they affect separate processes. To answer this, future experiments will need to perturb both pathways and compare their effects on each other and on the same developmental features.

Notch signalling has an evolutionarily conserved role in establishing tissue asymmetry [46]. In *Drosophila*, it regulates ommatidial rotation in the eye and planar polarity of sensory bristles [47]. In vertebrates, it establishes left–right asymmetry [48], and guides the orientation and patterning of mechanosensory hair cells [49,50]. Our study examined this mechanism in the developing mouse lower incisor. Notch signalling and epithelial–mesenchymal interactions are shared features of ectodermal appendage development [2], but our data do not show whether Notch-dependent differences in cell proliferation also occur in other appendages. It remains unclear whether teeth with different patterns of enamel distribution use similar regional differences in epithelial cell-cycle progression, or whether the mechanism we identified is specific to the labial–lingual organization of the mouse incisor. Comparative studies of different tooth classes and species, as well as other asymmetrically growing epithelia, will help determine how broadly this mechanism applies. Therefore, our findings identify a mechanism that contributes to early asymmetry in the mouse lower incisor. They also suggest a testable principle where local differences in signalling may direct growth by changing cell-cycle progression in specific regions.

Interestingly, epithelial-specific deletion of *Notch2* did not phenocopy pharmacological Notch inhibition. Instead, loss of *Notch2* was accompanied by upregulation of other Notch receptors, ligands, and downstream target genes, including *Notch1*, *Jag1*, *Dll1*, *Hes1*, *Hey1*, and *Hey2*. These transcriptional changes suggest a potential compensatory response within the Notch signalling network that contributes to the maintenance of the overall epithelial proliferation and morphology. This finding highlights the robustness and redundancy of the Notch signalling network, consistent with its established role as a stabilising element in developmental systems [51,52]. Such multilayered compensation might be in place to ensure reliable morphogenesis despite perturbations to individual pathway components.

No morphological modifications were detected during the early stages of incisor asymmetry, but changes may appear later. Indeed, deleting Notch2 from the epithelium leads to shorter postnatal incisors and defects in epithelial integrity and enamel formation [14,53]. Other Notch signals may compensate for the loss of Notch2 early in development, while Notch2 remains important for later incisor growth and homeostasis. Further studies are needed to determine when these changes first appear.

Beyond development, these findings have implications for regenerative medicine and tissue engineering [54]. Understanding how Notch integrates spatial patterning cues with cell-cycle regulation may inform strategies to engineer asymmetric tissues, reconstruct ectodermal organs, and direct stem cell differentiation in vitro. Moreover, dysregulation of Notch signalling and proliferative control is implicated in a wide range of epithelial disorders, including congenital malformations and carcinomas [10,55,56]. Elucidating the developmental mechanisms that couple spatial information to growth control may therefore provide insights into both disease pathogenesis and therapeutic intervention.

## 5. Conclusions

Our study demonstrates that asymmetric incisor growth is driven by regional differences in cell-cycle kinetics between the labial and lingual epithelia. We identify Notch signalling as an important regulator of this process, linking spatial patterning to proliferative behaviour. The reduced expression of cell-cycle regulators *Ccnd1* and *Cdk6* following pharmacological Notch inhibition further supports an association between Notch activity and cell-cycle regulation. Furthermore, the upregulation of other Notch pathway components after epithelial-specific Notch2 deletion suggests the existence of a compensatory network, that may preserve the directional epithelial growth and proper morphogenesis. More broadly, our findings provide a framework for understanding how conserved signalling pathways translate molecular asymmetry into tissue architecture, a principle that is likely to operate across diverse ectodermal appendages.

## Data Availability

The RNA sequencing data has been uploaded on the SRA NCBI database under the Bioproject PRJNA1542980 and submission number SUB16554652. The rest of the data that support the findings of this study are available from the corresponding author upon reasonable request.

## Supplementary Materials

The following supporting information can be downloaded at: https://www.mdpi.com/article/10.3390/cells15211947/s1, Figure S1: Immunohistochemistry reveals the involvement of Notch2 receptor in the labial epithelium during asymmetry generation.

## References

[1] Jiménez-Rojo L., Granchi Z., Graf D., Mitsiadis T.A. (2012). Stem Cell Fate Determination during Development and Regeneration of Ectodermal Organs. Front. Physiol..

[2] Pispa J., Thesleff I. (2003). Mechanisms of Ectodermal Organogenesis. Dev. Biol..

[3] Chuong C., Wu P., Plikus M., Jiang T., Bruce Widelitz R. (2005). Engineering Stem Cells into Organs: Topobiological Transformations Demonstrated by Beak, Feather, and Other Ectodermal Organ Morphogenesis. Curr. Top. Dev. Biol..

[4] Catón J., Luder H.-U., Zoupa M., Bradman M., Bluteau G., Tucker A.S., Klein O., Mitsiadis T.A. (2009). Enamel-Free Teeth: Tbx1 Deletion Affects Amelogenesis in Rodent Incisors. Dev. Biol..

[5] Li D., Wang X., Yao L., Jing H., Qin T., Li M., Zhang S., Chen Z., Zhang L. (2021). Sox2 Controls Asymmetric Patterning of Ameloblast Lineage Commitment by Regulation of FGF Signaling in the Mouse Incisor. J. Mol. Histol..

[6] Pouyet L., Mitsiadis T.A. (2000). Dynamic Lunatic Fringe Expression Is Correlated with Boundaries Formation in Developing Mouse Teeth. Mech. Dev..

[7] Mucchielli M., Mitsiadis T.A. (2000). Correlation of Asymmetric Notch2 Expression and Mouse Incisor Rotation. Mech. Dev..

[8] Kenji M. (2020). Notch Signaling. Dev. Growth Differ..

[9] Kopan R. (2012). Notch Signaling. Cold Spring Harb. Perspect. Biol..

[10] Andersson E.R., Sandberg R., Lendahl U. (2011). Notch Signaling: Simplicity in Design, Versatility in Function. Development.

[11] Mitsiadis T.A., Graf D., Luder H., Gridley T., Bluteau G. (2010). BMPs and FGFs Target Notch Signalling via Jagged 2 to Regulate Tooth Morphogenesis and Cytodifferentiation. Development.

[12] Mitsiadis T.A., Lardelli M., Lendahl U., Thesleff I. (1995). Expression of Notch 1, 2 and 3 Is Regulated by Epithelial-Mesenchymal Interactions and Retinoic Acid in the Developing Mouse Tooth and Associated with Determination of Ameloblast Cell Fate. J. Cell Biol..

[13] Mitsiadis T.A., Regaudiat L., Gridley T. (2005). Role of the Notch Signalling Pathway in Tooth Morphogenesis. Arch. Oral Biol..

[14] Jheon A.H., Prochazkova M., Meng B., Wen T., Lim Y.J., Naveau A., Espinoza R., Cox T.C., Sone E.D., Ganss B. (2016). Inhibition of Notch Signaling during Mouse Incisor Renewal Leads to Enamel Defects. J. Bone Miner. Res..

[15] Mustonen T., Tümmers M., Mikami T., Itoh N., Zhang N., Gridley T., Thesleff I. (2002). Lunatic Fringe, FGF, and BMP Regulate the Notch Pathway during Epithelial Morphogenesis of Teeth. Dev. Biol..

[16] Felszeghy S., Suomalainen M., Thesleff I. (2010). Notch Signalling Is Required for the Survival of Epithelial Stem Cells in the Continuously Growing Mouse Incisor. Differentiation.

[17] Harada H., Kettunen P., Jung H., Mustonen T., Wang Y.A., Thesleff I. (1999). Localization of Putative Stem Cells in Dental Epithelium and Their Association with Notch and FGF Signaling. J. Cell Biol..

[18] Mitsiadis T.A., Pagella P., Gomes Rodrigues H., Tsouknidas A., Ramenzoni L.L., Radtke F., Mehl A., Viriot L. (2023). Notch Signaling Pathway in Tooth Shape Variations throughout Evolution. Cells.

[19] Dassule H.R., Lewis P., Bei M., Maas R., McMahon A.P. (2000). Sonic Hedgehog Regulates Growth and Morphogenesis of the Tooth. Development.

[20] Yang X., Klein R., Tian X., Cheng H.-T., Kopan R., Shen J. (2004). Notch Activation Induces Apoptosis in Neural Progenitor Cells through a P53-Dependent Pathway. Dev. Biol..

[21] Fre S., Hannezo E., Sale S., Huyghe M., Lafkas D., Kissel H., Louvi A., Greve J., Louvard D., Artavanis-Tsakonas S. (2011). Notch Lineages and Activity in Intestinal Stem Cells Determined by a New Set of Knock-in Mice. PLoS ONE.

[22] Juuri E., Saito K., Ahtiainen L., Seidel K., Tummers M., Hochedlinger K., Klein O.D., Thesleff I., Michon F. (2012). Sox2+ Stem Cells Contribute to All Epithelial Lineages of the Tooth via Sfrp5+ Progenitors. Dev. Cell.

[23] Shroff N.P., Xu P., Kim S., Shelton E.R., Gross B.J., Liu Y., Gomez C.O., Ye Q., Drennon T.Y., Hu J.K. (2024). Proliferation-Driven Mechanical Compression Induces Signalling Centre Formation during Mammalian Organ Development. Nat. Cell Biol..

[24] Du W., Hu J.K.H., Du W., Klein O.D. (2017). Lineage Tracing of Epithelial Cells in Developing Teeth Reveals Two Strategies for Building Signaling Centers. J. Biol. Chem..

[25] Ankamreddy H., Thawani A., Birol O., Zhang H., Groves A.K. (2023). *Foxi3^GFP^* and *Foxi3^CreER^* Mice Allow Identification and Lineage Labeling of Pharyngeal Arch Ectoderm and Endoderm, and Tooth and Hair Placodes. Dev. Dyn..

[26] Sun Z., Yu W., Sanz Navarro M., Sweat M., Eliason S., Sharp T., Liu H., Seidel K., Zhang L., Moreno M. (2016). Sox2 and Lef-1 Interact with Pitx2 to Regulate Incisor Development and Stem Cell Renewal. Development.

[27] Jussila M., Aalto A., Navarro M.S., Shirokova V., Balic A., Kallonen A., Ohyama T., Groves A., Mikkola M.L., Thesleff I. (2015). Suppression of Epithelial Differentiation by Foxi3 Is Essential for Molar Crown Patterning. Development.

[28] Yoshinaga K., Yasue A., Mitsui S.N., Minegishi Y., Oyadomari S., Imoto I., Tanaka E. (2023). Effects of Wnt10a and Wnt10b Double Mutations on Tooth Development. Genes.

[29] Nowakowski R.S., Lewin S.B., Miller M.W. (1989). Bromodeoxyuridine Immunohistochemical Determination of the Lengths of the Cell Cycle and the DNA-Synthetic Phase for an Anatomically Defined Population. J. Neurocytol..

[30] Lehal R., Zaric J., Vigolo M., Urech C., Frismantas V., Zangger N., Cao L., Berger A., Chicote I., Loubéry S. (2020). Pharmacological Disruption of the Notch Transcription Factor Complex. Proc. Natl. Acad. Sci. USA.

[31] Hatakeyama M., Opitz L., Russo G., Qi W., Schlapbach R., Rehrauer H. (2016). SUSHI: An Exquisite Recipe for Fully Documented, Reproducible and Reusable NGS Data Analysis. BMC Bioinform..

[32] Bray N.L., Pimentel H., Melsted P., Pachter L. (2016). Near-Optimal Probabilistic RNA-Seq Quantification. Nat. Biotechnol..

[33] Frankish A., Diekhans M., Jungreis I., Lagarde J., Loveland J.E., Mudge J.M., Sisu C., Wright J.C., Armstrong J., Barnes I. (2021). GENCODE 2021. Nucleic Acids Res..

[34] Love M.I., Huber W., Anders S. (2014). Moderated Estimation of Fold Change and Dispersion for RNA-Seq Data with DESeq2. Genome Biol..

[35] Yu G., Wang L.-G., Han Y., He Q.-Y. (2012). ClusterProfiler: An R Package for Comparing Biological Themes Among Gene Clusters. OMICS.

[36] Berg S., Kutra D., Kroeger T., Straehle C.N., Kausler B.X., Haubold C., Schiegg M., Ales J., Beier T., Rudy M. (2019). Ilastik: Interactive Machine Learning for (Bio)Image Analysis. Nat. Methods.

[37] Schindelin J., Arganda-Carreras I., Frise E., Kaynig V., Longair M., Pietzsch T., Preibisch S., Rueden C., Saalfeld S., Schmid B. (2012). Fiji: An Open-Source Platform for Biological-Image Analysis. Nat. Methods.

[38] Martí-Clúa J. (2023). Methods for Inferring Cell Cycle Parameters Using Thymidine Analogues. Biology.

[39] Kyrylkova K., Kyryachenko S., Biehs B., Klein O., Kioussi C., Leid M. (2012). BCL11B Regulates Epithelial Proliferation and Asymmetric Development of the Mouse Mandibular Incisor. PLoS ONE.

[40] Cotton M.J., Ariel P., Chen K., Walcott V.A., Dixit M., Breau K.A., Hinesley C.M., Kedziora K.M., Tang C.Y., Zheng A. (2024). An in Vitro Platform for Quantifying Cell Cycle Phase Lengths in Primary Human Intestinal Epithelial Cells. Sci. Rep..

[41] Snow M.H.L. (1977). Gastrulation in the Mouse: Growth and Regionalization of the Epiblast. Development.

[42] Haque A., Hata M., Marchuk K., Marangoni P., Green J., Klein O.D. (2026). Asymmetry of Sonic Hedgehog Expression Is Necessary for Early Incisor Tooth Morphogenesis. Development.

[43] Young T., Deschamps J. (2009). Chapter 8 Hox, Cdx, and Anteroposterior Patterning in the Mouse Embryo. Curr. Top. Dev. Biol..

[44] Das D., Lanner F., Main H., Andersson E.R., Bergmann O., Sahlgren C., Heldring N., Hermanson O., Hansson E.M., Lendahl U. (2010). Notch Induces Cyclin-D1-Dependent Proliferation during a Specific Temporal Window of Neural Differentiation in ES Cells. Dev. Biol..

[45] Joshi I., Minter L.M., Telfer J., Demarest R.M., Capobianco A.J., Aster J.C., Sicinski P., Fauq A., Golde T.E., Osborne B.A. (2009). Notch Signaling Mediates G1/S Cell-Cycle Progression in T Cells via Cyclin D3 and Its Dependent Kinases. Blood.

[46] Hermann G.J., Leung B., Priess J.R. (2000). Left-Right Asymmetry in *C. Elegans* Intestine Organogenesis Involves a LIN-12/Notch Signaling Pathway. Development.

[47] Strutt D., Johnson R., Cooper K., Bray S. (2002). Asymmetric Localization of Frizzled and the Determination of Notch-Dependent Cell Fate in the Drosophila Eye. Curr. Biol..

[48] Przemeck G.K.H., Heinzmann U., Beckers J., Hrabé de Angelis M. (2003). Node and Midline Defects Are Associated with Left-Right Development in *Delta1* Mutant Embryos. Development.

[49] Jacobo A., Dasgupta A., Erzberger A., Siletti K., Hudspeth A.J. (2019). Notch-Mediated Determination of Hair-Bundle Polarity in Mechanosensory Hair Cells of the Zebrafish Lateral Line. Curr. Biol..

[50] Kozak E.L., Palit S., Miranda-Rodríguez J.R., Janjic A., Böttcher A., Lickert H., Enard W., Theis F.J., López-Schier H. (2020). Epithelial Planar Bipolarity Emerges from Notch-Mediated Asymmetric Inhibition of Emx2. Curr. Biol..

[51] Mase S., Shitamukai A., Wu Q., Morimoto M., Gridley T., Matsuzaki F. (2021). Notch1 and Notch2 Collaboratively Maintain Radial Glial Cells in Mouse Neurogenesis. Neurosci. Res..

[52] Romero-Wolf M., Shin B., Zhou W., Koizumi M., Rothenberg E.V., Hosokawa H. (2020). Notch2 Complements Notch1 to Mediate Inductive Signaling That Initiates Early T Cell Development. J. Cell Biol..

[53] Lamprou A., Porcheri C., Mitsiadis T.A. (2025). Notch2 Deletion Compromises Epithelial Integrity and Enamel Formation in Rodent Incisors. Cells.

[54] Tiemeijer L.A., Sanlidag S., Bouten C.V.C., Sahlgren C.M. (2022). Engineering Tissue Morphogenesis: Taking It up a Notch. Trends Biotechnol..

[55] Siebel C., Lendahl U. (2017). Notch Signaling in Development, Tissue Homeostasis, and Disease. Physiol. Rev..

[56] Andersson E.R., Lendahl U. (2014). Therapeutic Modulation of Notch Signalling—Are We There Yet?. Nat. Rev. Drug Discov..

