# Supplementary material for: Notch Signalling Controls Differential Proliferation During Rotation and Asymmetric Morphogenesis of Mouse Incisors
Source: Cells. 2026 Oct 26;15(21):1947. doi: 10.3390/cells15211947 (PMC13762384; doi:10.3390/cells15211947)
Supplement: Supplementary file 1 [file cells-15-01947-s001.zip › cells-4604998-supplementary.pdf]

Notch Signalling Controls Differential Proliferation During Rotation and Asymmetric Morphogenesis of Mouse Incisors

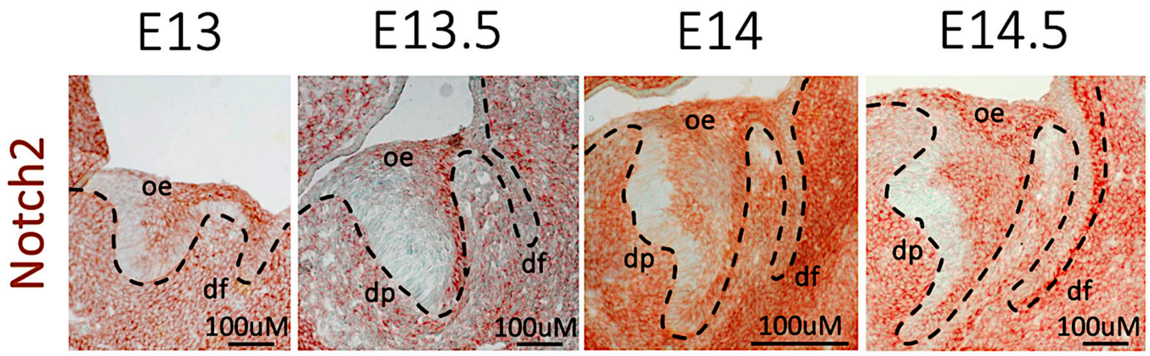

Figure S1. Immunohistochemistry reveals the involvement of Notch2 receptor in the labial epithelium during asymmetry generation. Immunohistochemistry for Notch2 in E13-E14.5 incisors. Black dashed lines outline the incisor epithelium. Stainings were repeated in sections from 3 independent biological samples. Abbreviations: df, dental furrow; dp, dental pulp; oe, oral epithelium.
